# Autism spectrum disorder with extreme behavioral dysregulation: pharmacogenetic challenges and family burden

**DOI:** 10.1192/j.eurpsy.2026.11157

**Published:** 2026-07-31

**Authors:** C. Garcia Cerdan, M. Ligero Argudo, I. M. Peso Navarro, M. L. García Ullán, C. Martín Gómez, J. Pérez Rodríguez, C. Munaiz Cossio, R. K. González Bolaños, J. I. de la Iglesia Larrad, C. Payo Rodríguez, A. I. Mitadiel Velasco

**Affiliations:** 1 Psychiatry, University Clinical Hospital; 2 Psychiatry, Institute of Biomedical Research of Salamanca (IBSAL); 3 Psychology, University Clinical Hospital, Salamanca, Spain

## Abstract

**Introduction:**

Severe forms of autism spectrum disorder (ASD) may present with extremely intense behavioral disturbances that are resistant to treatment, with significant clinical and social consequences. The limited efficacy of psychopharmacological interventions, together with pharmacogenetic restrictions, often results in only partial symptom control. These situations generate an extraordinary burden on primary caregivers.

**Objectives:**

To present a case of severe ASD with extreme behavioral disturbances and poor response to multiple treatments, highlighting the profound family impact and the limitations associated with pharmacogenetics.

**Methods:**

This work is based on the detailed analysis of a clinical case admitted to the acute psychiatric unit of the University Hospital of Salamanca, complemented with a review of the relevant literature.

Case description: A 21-year-old male with severe ASD and multiple prior psychiatric hospitalizations was admitted due to episodes of extreme agitation, self-injury, and aggression. The severity of the symptoms required exceptional protective measures, including the use of a boxing helmet to prevent cranial trauma and boxing gloves to reduce injury from striking.

Despite these precautions, the patient developed a stress fracture of the metatarsal. Pharmacological management was further complicated by a deficient metabolic profile, with limited response to several drugs and the need for continuous adjustments. The family, as the main source of support, became overwhelmed, experiencing significant emotional and physical exhaustion. Following treatment adjustments with risperidone and topiramate, partial improvement was achieved, and the patient was referred to a specialized regional unit for further care.

**Results:**

This case illustrates the complexity of managing severe behavioral disturbances in ASD. Treatment resistance and pharmacogenetic limitations compromise therapeutic effectiveness. The impact on the family is devastating, with exposure to risk, emotional exhaustion, feelings of helplessness in the face of violence, and continuous physical and psychological strain. Clinical management should not only focus on the patient but also actively include support and guidance for caregivers, whose role is essential yet extremely vulnerable.

**Conclusions:**

- Severe behavioral disturbances in ASD can reach extreme intensity, with high risk of self-injury and aggression.
- Pharmacogenetic limitations further restrict therapeutic options.
- The family impact is profound, leading to emotional, physical, and social overload that exceeds typical caregiving capacities.
- Strengthening caregiver support and designing comprehensive strategies that acknowledge their fundamental role is imperative.

**Disclosure of Interest:**

None Declared

